# Continuity in a VA Patient-Centered Medical Home Reduces Emergency Department Visits

**DOI:** 10.1371/journal.pone.0096356

**Published:** 2014-05-27

**Authors:** Krisda H. Chaiyachati, Kirsha Gordon, Theodore Long, Woody Levin, Ali Khan, Emily Meyer, Amy Justice, Rebecca Brienza

**Affiliations:** 1 Center of Excellence in Primary Care Education, VA Connecticut Healthcare System, West Haven, Connecticut, United States of America; 2 Department of Internal Medicine, Yale University School of Medicine, New Haven, Connecticut, United States of America; 3 VA Connecticut Healthcare System, West Haven, Connecticut, United States of America; 4 Robert Wood Johnson Clinical Scholars Program, Yale School of Medicine, New Haven, Connecticut, United States of America; 5 Evergreen Design, Guilford, Connecticut, United States of America; 6 Iora Health, Collective Primary Care, Brooklyn, New York, United States of America; Cardiff University, United Kingdom

## Abstract

**Background:**

One major goal of the Patient-Centered Medical Home (PCMH) is to improve continuity of care between patients and providers and reduce the utilization of non-primary care services like the emergency department (ED).

**Objective:**

To characterize continuity under the Veterans Health Administration’s PCMH model – the Patient Aligned Care Team (PACT), at one large Veterans Affair’s (VA’s) primary care clinic, determine the characteristics associated with high levels of continuity, and assess the association between continuity and ED visits.

**Design:**

Retrospective, observational cohort study of patients at the West Haven VA (WHVA) Primary Care Clinic from March 2011 to February 2012.

**Patients:**

The 13,495 patients with established care at the Clinic, having at least one visit, one year before March 2011.

**Main Measures:**

Our exposure variable was continuity of care –a patient seeing their assigned primary care provider (PCP) at each clinic visit. The outcome of interest was having an ED visit.

**Results:**

The patients encompassed 42,969 total clinic visits, and 3185 (24%) of them had 15,458 ED visits. In a multivariable logistic regression analysis, patients with continuity of care – at least one visit with their assigned PCP – had lower ED utilization compared to individuals without continuity (adjusted odds ratio [AOR] 0.54; 95% CI: 0.41, 0.71), controlling for frequency of primary care visits, comorbidities, insurance, distance from the ED, and having a trainee PCP assigned. Likewise, the adjusted rate of ED visits was 544/1000 person-year (PY) for patients with continuity vs. 784/1000 PY for patients without continuity (p = 0.001). Compared to patients with low continuity (<33% of visits), individuals with medium (33–50%) and high (>50%) continuity were less likely to utilize the ED.

**Conclusions:**

Strong continuity of care is associated with decreased ED utilization in a PCMH model and improving continuity may help reduce the utilization of non-primary care services.

## Introduction

The U.S. healthcare system has become disjointed, a challenge for achieving patient-centered care [1]–[3]. The patient-centered medical home (PCMH) model was conceived almost a half-century ago to facilitate greater integration and continuity**,** but did not gain prominence until a 2007 joint collaborative established a series of principles now used to define the medical home [4]–[6]. After adopting these principles and successfully piloting PCMH care at one Veterans Affairs (VA) primary care clinic, the Veteran’s Health Administration began transitioning all of its primary care clinics towards a PCMH structure under the title, the Patient Aligned Care Team (PACT), in 2008 [7]–[12].

One hallmark goal of PACT involves improved continuity of care between a patient and their primary care provider (PCP), partly because poor continuity is believed to contribute to the inappropriate use of healthcare services like emergency departments (ED) [13]–[17]. As a result, the VA assigned care teams to each patient within PACT and implemented efforts to increase patient access to providers. VA clinics, often serving as training sites for health professionals, face additional challenges towards achieving strong continuity because trainees rotate through inconsistently, making appointment scheduling difficult.

Many observational and pilot studies indicate PCMH models can decrease care fragmentation, reduce utilization of healthcare resources and costs, and decrease ED visits [9]–[12], [18]–[22]. These observations of PCMH implementation, however, do not clearly characterize the degrees of continuity achieved nor do they analyze associations between continuity and ED utilization. Hypothesizing that patients with high continuity of care levels would visit the ED less, we sought to model patient and clinic level factors associated with continuity and assess the association between high levels of continuity and ED visits in the year after the 2011 implementation of PACT at Connecticut’s West Haven VA (WHVA).

## Methods

### Ethical Review

Institutional review boards at the VA Connecticut Healthcare System (VACHS) and Yale University School of Medicine (New Haven, CT) approved this study. No informed consent was taken. Records and information were anonymous and de-identified prior to analysis.

### Cohort

We performed a retrospective analysis of patients who visited the WHVA primary care clinic one year after restructuring under PACT: March 1, 2011 to February 29, 2012. To minimize confounding by different utilization patterns of new WHVA patients, we extracted electronic medical records of patients with established WHVA care, defined as at least one primary care visit one year before restructuring (March 1, 2010 to February 28, 2011). Patient data was extracted from the Veterans Health Information Systems and Technology Architecture and processed by the 2010 Microsoft Structured Query Language Server using Cache Structured Query Language and Open Database Connectivity connections.

### Setting

WHVA primary care services consist of routine primary care clinics and a separate women’s clinic allocated for female Veteran care. Though VACHS consists of the WHVA, the Newington campus, and six community-based outpatient clinics (CBOCs) throughout the state, this analysis is limited to the WHVA’s Primary Care Clinic. The exclusion of the Newington campus and CBOCs was deliberate because of the physical distance and clinic level factors that might influence how individuals utilized the WHVA ED, the only VA ED in the VACHS network.

### PACT Team Description

Each patient is assigned to a PACT team, which consists of a) a licensed PCP – physician (MD or DO), nurse practitioner (NP), or physician assistant (PA); b) a registered nurse (RN); and c) a medical assistant. Additional on-site staff members include pharmacists, dieticians, social workers, care coordinators, health psychologists, and specialty medical or surgical consultants.

During the study period, over 40 internal medicine resident trainees and two NP fellow trainees were supervised by PCPs and assigned to PACT teams as part of their outpatient training requirements. Moreover, beginning June 2011 and beyond the study period end date, the WHVA had two of its PACT teams participating in the Center of Excellence in Primary Care Education, a training model designed to prepare trainees from different health professions in providing coordinated, interprofessional, outpatient care [23]. The WHVA had 13 clinical, full-time equivalent providers during the study period.

### Continuity

We defined continuity as a patient seeing their assigned PCP or trainee (e.g. resident physician or NP).

**Figure 1:**
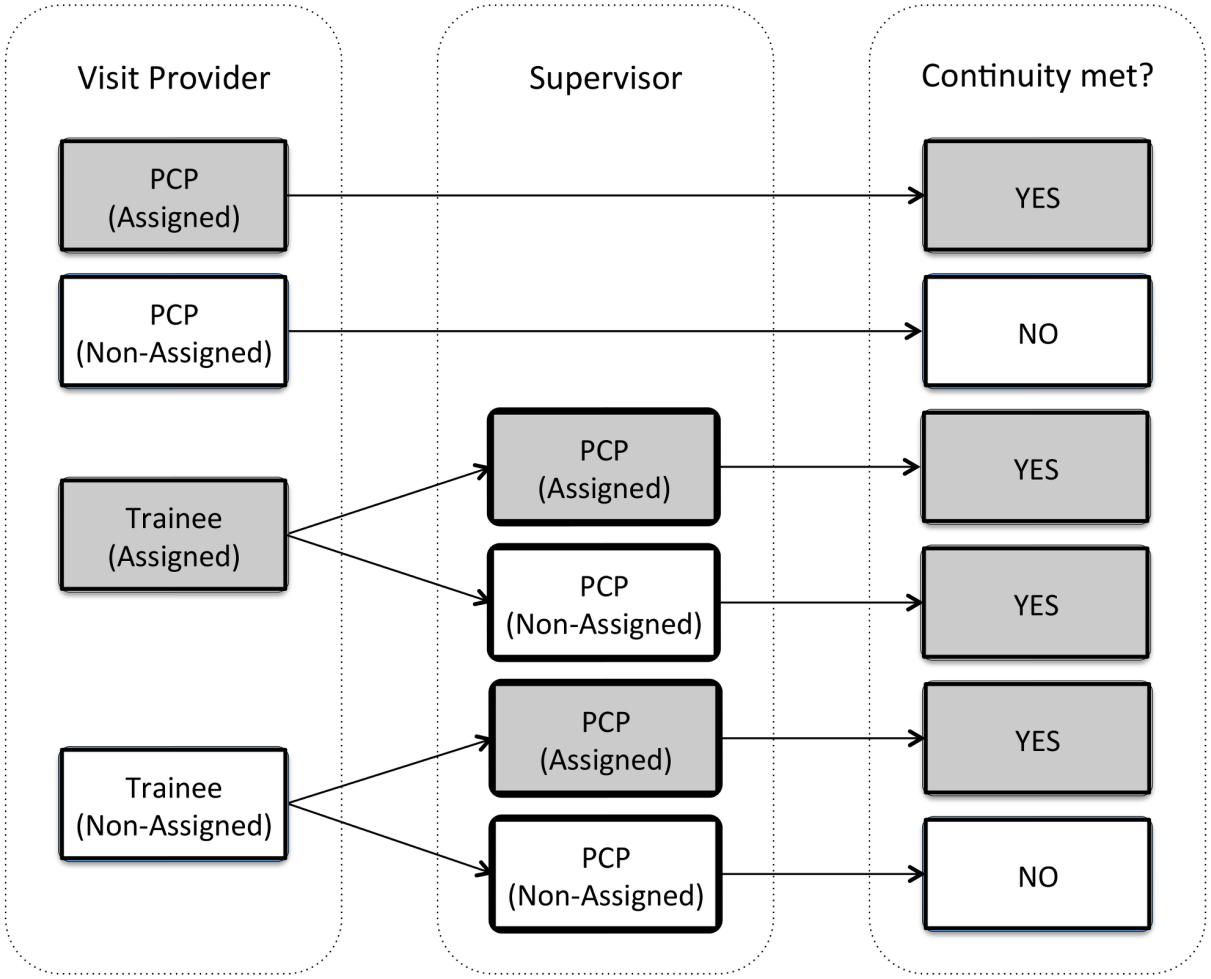
Potential Patient Visit Interactions with a Provider and/or Trainee and the Relationship with Continuity. PCP: primary care provider. Grey boxes indicate visits with continuity of care and white boxes indicate visits without continuity.

Two assignment scenarios exist for patients – *Assignment A*: a patient is assigned to a PCP without a trainee also managing their outpatient care or *Assignment B*: a patient is assigned to a trainee and PCP who supervises that trainee. No patient is ever assigned to a trainee provider without also being assigned to a PCP, since trainees require supervision by a licensed PCP. Patients who are evaluated by trainees require supervision by a PCP during the same visit. Therefore, two visit scenarios exist for patients – a PCP without the additional care of a trainee evaluates the patients or a trainee who is supervised by a PCP evaluates the patient. In the latter scenario, we defined continuity as the patient seeing either their assigned trainee or a separate trainee supervised by the patient’s assigned PCP. When evaluating continuity, we accounted for these various combinations of providers who may see a patient during a given visit (**Figure 1**); and through the electronic medical record we could account for any patient-PCP or patient-trainee reassignments that might have occurred during the study period.

After determining whether each visit had continuity or not, we calculated a continuity index for each patient based on the Usual Provider Continuity (UPC) definition, which calculates the percentage of the number of visits to the assigned provider divided by the total number of visits [24], [25]. Levels of continuity were created based on the index into low (<33% of visits/year had continuity), medium (33–50% of visits/year), and high (>50% of visits/year).

### ED Visits

The main outcome of interest was ED visits during the study period by each study patient. We controlled for distance by using the zip code difference between the patient’s address and the WHVA as a proxy. We also determined visit times and day of the week (i.e. weekend versus weekday).

### Covariates and Potential Confounders

We examined baseline demographics including age, sex, and race, categorized by the patient as white non-Hispanic, black, Hispanic, or other race. Other covariates of interest were percent service connection – degree of health compensation benefit paid for a designated condition related to injury or illness incurred or aggravated by active military service, period of military service, and presence of outside health insurance. Comorbidity data based on ICD-9 codes were collected, including mental illness (having major depression, bipolar disorder, post-traumatic stress disorder, or schizophrenia), substance abuse (drug abuse or alcohol abuse and dependence), myocardial infarction and coronary artery disease, congestive heart failure, diabetes mellitus, hypertension, peripheral vascular disease, pulmonary diseases, stroke, hyperlipidemia, flu and obesity [15], [16]. Linking particular ICD-9 codes with comorbidity diagnoses have been described previously [26], [27]. We derived smoking prevalence from Veterans Affairs Health Factors database, a computerized clinical provider reminder and reporting system that periodically reminds clinicians to perform assessments of tobacco and alcohol use, then records the results of these structured interviews. Individuals who sparingly visited the WHVA were believed to use the WHVA ED services differently than those who visit routinely; therefore, we tracked the frequency of primary care visits, defining “low” primary care users as less than three primary care visits per year, while “high” primary care users had at least three [15].

### Statistical Analysis

We compared demographic characteristics using the student t-test for normally distributed variables, Wilcoxon-Mann Whitney for non-parametric variables, and the chi-square for categorical variables. Bivariate analyses determined the impact demographic data may have on the association between continuity and ED use. For individuals with complete data, multivariable logistic regression models were constructed to assess the relationship between continuity and ED use. In addition, rates of ED visits/1000 person-year (PY) were calculated, adjusted for similar variables as in the multivariable logistic regression. To assess the impact levels of continuity have on ED utilization, models were restricted to individuals who had at least one continuity visit. All models were run unadjusted and then adjusted for the following variables: age, gender, comorbidities, primary care user level, outside insurance, percent service connection, service period, zip-code differences, and having a trainee provider assigned to a patient versus not. In the sensitivity analysis, we calculated stratum-specific odds ratios for the number of primary care visits during the study period instead of categorizing this variable into “low” and “high” primary care users. A p-value of <0.05 was used to determine statistical significance. Statistical analyses were performed using SAS version 9.2 (SAS Institute Inc., North Carolina).

## Results

Our cohort included 13,495 individuals who totaled 42,969 unique primary care clinic visits. Among those, 3185 (24%) had an ED visit; accounting for 15,458 ED visits.

The cohort was primarily composed of white, male Veterans with a median age of 69 years (**Table 1**). The median number of primary care visits was 2 (IQR 1–4). Veterans who had no continuity with their assigned PCPs totaled 364 (3%) individuals. The mean UPC index was 87% (median 1.0; IQR 0.89–1.0)**.** The percentage of low primary care users (<3 visits) was 56% while the percentage of high primary care users (≥3 visits) was 44%. Nineteen percent of patients had a trainee assigned to their care.

**Table 1:** Patient Demographics by Continuity and Stratification by Continuity of Care Levels.

	Overall (N = 13,495)	Continuity			Among Patients with ≥1 Continuity Visit			
Variables	%	No (n = 364)	Yes (n = 13131)	p-value	<33% (n = 307)	33%–50% (n = 1277)	>50% (n = 11547)	p-value
**Age,** IQR	69 (61, 80)	60 (44, 77)	69 (61, 80)	<0.001	64 (51, 74)	65 (55, 77)	70 (62, 81)	<0.001
**Sex**				<0.001				<0.001
Female	5	20	4		10	10	4	
Male	95	80	96		90	90	96	
**Race** a				0.03				0.51
White	80	71	80		76	79	80	
Black	15	26	15		19	15	15	
Hispanics	3	3	3		2	4	3	
Other	2	0	2		3	2	2	
**Smoker**	19	26	18	<0.001	21	23	18	<0.001
**Substance use disorder**	8	9	8	0.21	11	10	7	<0.001
**Severe mental iIlness** b	10	14	10	0.01	19	15	9	<0.001
**Service connection (%)**								
0–25	11	11	11	1.00	11	10	11	0.65
26–50	7	9	6	0.07	11	9	6	<0.001
51–75	5	6	5	0.27	8	7	4	<0.001
76–100	7	12	7	0.003	15	11	7	<0.001
**Period of service**				<0.001				<0.001
Other^c^	2	3	2		3	2	2	
Korean	17	9	18		13	15	18	
Persian Gulf	9	27	9		18	13	8	
Post-Korean	10	6	10		6	8	10	
Post-Vietnam	8	15	8		14	14	7	
Vietnam Era	36	24	36		37	37	36	
World War II	17	16	17		9	12	18	
**Outside insurance (yes)**	69	52	70	<0.001	65	67	70	0.01
**Primary care user level**				<0.001				<0.001
Low (<3 visits/year)	56	71	55		0	16	61	
High (≥3 visits/year)	44	29	45		100	84	39	
**Comorbidities**								
MI/Coronary artery disease	23	13	23	<0.001	20	21	23	0.20
Congestive heart failure	3	2	3	0.08	5	6	3	<0.001
Diabetes mellitus	26	18	26	<0.001	26	31	25	<0.001
Hypertension	64	37	65	<0.001	57	65	66	0.01
Peripheral vascular disease	5	6	5	0.78	6	6	5	0.26
Pulmonary disorder/COPD	11	9	11	0.27	15	16	10	<0.001
Stroke/TIA	4	1	4	<0.001	6	5	4	0.08
Alcohol	6	5	6	0.76	8	7	6	0.05
Drug	3	6	3	<0.001	5	4	2	<0.001
Pneumonia	0.01	0	0.01	1.00	0	0	0.01	-
Schizophrenia	1	3	1	<0.001	2	1	1	0.03
Bipolar disorder	1	1	1	0.97	3	2	1	<0.001
Major Depression	3	4	3	0.07	5	6	2	<0.001
PTSD	6	6	6	0.55	12	8	5	<0.001
Hyperlipidemia	57	30	58	<0.001	50	54	58	<0.002
Influenza	33	21	33	<0.001	43	40	33	<0.001
Overweight/obesity	14	10	15	0.03	22	21	14	<0.001
**ED visits**	24	32	23	<0.001	50	38	21	<0.001
**No. of ED visits after hours on a weekday** d	22	27	23	0.34	23	23	23	0.99
**ED visit day** d				0.15				0.07
Weekday	78	83	78		73	81	78	
Weekend	22	17	22		27	19	22	
**No. ED visits** d				0.85				0.12
1	11	13	11		10	12	11	
2	24	21	25		21	21	26	
3	17	16	17		14	16	17	
4 or more	47	50	47		55	51	46	
**Assigned provider(s)**				<0.001				<0.001
PCP only	81	74	82		90	88	81	
Trainee & PCP	19	26	18		10	12	19	
**Zip code distance from ED**				0.11				<0.001
≤2	15	14	15		19	17	15	
3–5	11	10	11		12	13	11	
6–10	1	0.3	1		2	1	1	
11–50	18	15	18		14	16	19	
>50	55	60	55		53	52	55	

Abbreviations: IQR, interquartile range; MI, myocardial infarction; COPD, chronic obstructive pulmonary disease; TIA, transient ischemic attack; PTSD, post-traumatic stress disorder; ED, emergency department.

a Sixty-nine percent were missing this variable.

b Composite of major depression, bipolar, schizophrenia and PTSD.

c Because of the small number of veterans in these groups, they were combined: active military personnel, CAV/NPS, ChampVA spouse and children, non-Veteran humanitarian groups, merchant marines, and Tricare.

d Among ED visits that took place between Monday and Friday.

Individuals having at least one continuity visit were less likely to visit the ED compared to those lacking a single continuity visit (23% vs. 32%, p<0.001) (**Table 1**). They were less likely to actively smoke, have higher service connection, have severe mental illness, and abuse drugs. They were more likely to be white, have outside insurance, visit the primary care clinic more than three times in the year, served in Korea or Vietnam, have a diagnosis of coronary artery disease or a myocardial infarction, diabetes, hypertension, stroke or transient ischemic attack, hyperlipidemia, or obesity. Patients assigned to trainees were less likely to have continuity of care.

### Unadjusted Predictors of ED Utilization

Compared with individuals who did not use the ED (**Table 2**), ED users were significantly (p<0.05 for all variables) younger, female, smoke, use substances, have a severe mental illness, have a high degree of service connection, more frequently visited the primary care clinic, and have the following comorbidities: congestive heart failure, a pulmonary disorder, stroke, flu, and obesity. In contrast, ED users were less likely to have outside insurance. In the unadjusted logistic regression model, having at least one primary care visit with continuity was associated with a 36% reduction in visits to the ED (odds ratio [OR] 0.64; 95% confidence interval [CI]: 0.51, 0.80). The unadjusted rate of ED visits for patients with continuity vs. none was 204/1000 PY (95% CI: 141, 294) vs. 266/1000 PY (95% CI: 222, 319) (p = 0.004).

**Table 2:** Patient Demographics by Emergency Department Visits.

	ED Visits		
Variables	No, n = 10310	Yes, n = 3185	p-value
**Age,** IQR	70 (62, 80)	65 (56, 78)	<0.001
**Sex**			<0.001
Female	4	7	
Male	96	93	
**Race** a			<0.001
White	81	77	
Black	14	19	
Hispanics	3	3	
Other	2	2	
**Smoker (yes)**	16	25	<0.001
**Substance use disorder (yes)**	6	14	<0.001
**Severe mental illness** b **(yes)**	8	16	<0.001
**Service connection (%)**			
0–25	11	11	0.67
26–50	6	8	<0.001
51–75	4	6	<0.001
76–100	6	12	<0.001
**Period of service**			<0.001
Other^c^	2	2	
Korean	19	13	
Persian Gulf War	9	11	
Post-Korean War Period	10	7	
Post-Vietnam War Period	7	13	
Vietnam War	35	39	
World War II	17	15	
**Outside insurance status (yes)**	72	61	<0.001
**Primary care user level**			<0.001
Low (<3 visits/year)	64	30	
High (≥3 visits/year)	36	70	
**Continuity**	98	96	<0.001
<33%	2	5	<0.001
33%–50%	8	16	
>50%	91	79	
**Comorbidities**			
Coronary artery disease/MI	23	23	0.91
Congestive heart failure	2	6	<0.001
Diabetes mellitus	24	29	<0.001
Hypertension	65	63	0.15
Peripheral vascular disease	5	6	0.004
Pulmonary disorder/COPD	9	17	<0.001
Stroke/TIA	4	6	<0.001
Alcohol	5	10	<0.001
Drug	2	6	<0.001
Pneumonia	0	0.03	0.24
Schizophrenia	1	2	<0.001
Bipolar disorder	1	3	<0.001
Major Depression	2	5	<0.001
PTSD	5	8	<0.001
Hyperlipidemia	58	52	<0.001
Influenza	31	41	<0.001
Overweight/obesity	14	17	<0.001
**Primary care visits,** IQR	2 (1, 3)	4 (2, 6)	<0.001
**Continuity visits,** IQR	2 (1, 3)	3 (2, 4)	<0.001
**Assigned provider(s)**			<0.001
PCP only	82	79	
Trainee & PCP	18	21	
**Zip code distance from ED**			<0.001
≤2	13	20	
3–5	9	16	
6–10	1	2	
11–50	20	14	
>50	57	49	

Abbreviations: IQR, interquartile range; MI, myocardial infarction; COPD, chronic obstructive pulmonary disease; TIA, transient ischemic attack; PTSD, post-traumatic stress disorder; PCP, primary care provider.

a Sixty-nine percent (69%) were missing this variable.

b Composite of major depression, bipolar, schizophrenia and PTSD.

c Because of the small number of veterans in these groups, they were combined: active military personnel, CAV/NPS, ChampVA spouse and children, non-Veteran humanitarian groups, merchant marines, and Tricare.

### Adjusted Predictors of ED Utilization

**Figure 2:**
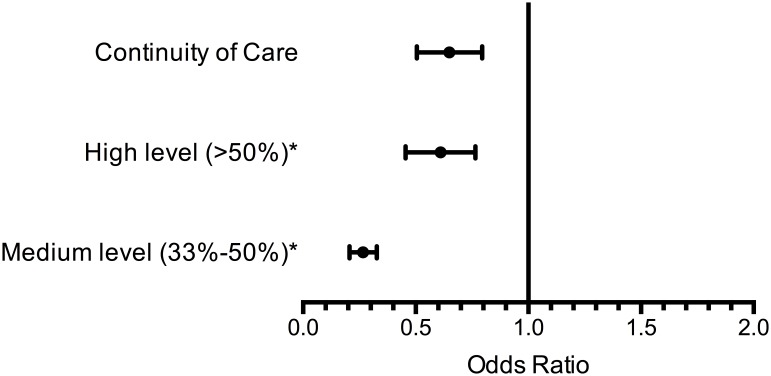
Adjusted Odds Ratio of Association Between ED Visit and Continuity of Care and Levels of Continuity. The first row illustrates the adjusted odds ratio between ED visits and continuity of care for individuals with at least one visit having continuity with their primary care provider. The second and third rows are the adjusted odds ratio for individuals who had high and medium levels of continuity and are compared to individuals with low levels of continuity, indicated by the asterisk (*).

In the adjusted multivariable logistic regression (**Table 3** **&** **Figure 2**), continuity reduced ED utilization by 46% (adjusted odds ratio [AOR] 0.54; 95% CI: 0.41, 0.71). Likewise, the rate of ED visits for patients with continuity, 544/1000 PY (95% CI: 277, 1106), was lower compared to the patients without continuity, 784/1000 PY (95% CI: 484, 1272) (p = 0.001). Older age (AOR 0.91; 95% CI: 0.84, 0.98), low primary care clinic use (AOR 0.27; 95% CI: 0.24, 0.29), lower (<50%) service connection level (AOR 0.68; 95% CI: 0.58, 0.80), living far from the WHVA (>11 zip codes: AOR 0.53; 95% CI: 0.48, 0.59), and having a trainee provider assigned (AOR 0.78; 95% CI: 0.69, 0.89) were associated with lower ED utilization. Predictive factors included comorbidities such as smoking (AOR 1.22; 95% CI: 1.09, 1.37), substance abuse disorder (AOR 1.73; 95% CI: 1.47, 2.03), severe mental illness (AOR 1.31; 95% CI: 1.13, 1.51), coronary artery disease (AOR 1.19; 95% CI: 1.06, 1.34), congestive heart failure (AOR 2.38; 95% CI: 1.91, 2.97), diabetes (AOR 1.17; 1.05, 1.31), pulmonary disorders (AOR 1.68; 95% CI: 1.47, 1.92), and stroke (AOR 1.55; 95% CI: 1.26, 1.91); and compared with Vietnam era Veterans, serving during the post-Vietnam era (AOR 1.36; 95% CI: 1.13, 1.64) or World War II (AOR 1.40; 95% CI: 1.11, 1.75).

**Table 3:** Association Between Continuity of Care and Emergency Department Utilization.

Rates of ED Utilization						
	*Unadjusted*			*Adjusted* a ^,^ b		
Variables	Visits/1000 PY (95% CI)	p-value		Visits/1000 PY (95% CI)		p-value
**No Continuity** c	266 (222, 319)	-		784 (484, 1272)		-
**Continuity**	204 (141, 294)	0.004		544 (277, 1106)		<0.001
*Odds of ED Utilization*						
	***Unadjusted***		***Adjusted*** a **^,^** b			
**Variables**	**OR (95% CI)**	**p-value**	**AOR (95% CI)**		**p-value**	
**Continuity**	0.64 (0.51, 0.80)	0.001	0.54 (0.41, 0.71)		<0.001	
**Continuity of care level**						
Low (<33%)^c^	1.00	-	-		-	
Medium (33–50%)	0.60 (0.46, 0.77)	<0.001	-		-	
High (>50%)	0.26 (0.21, 0.33)	<0.001	-		-	
**Age/10**	0.85 (0.83, 0.88)	<0.001	0.91 (0.84, 0.98)		0.02	
**Male**	0.64 (0.53, 0.76)	<0.001	0.93 (0.75, 1.15)		0.49	
**Comorbidities**						
Smoker	1.75 (1.59, 1.92)	<0.001	1.22 (1.09, 1.38)		0.001	
Substance use disorder	2.83 (2.49, 3.23)	<0.001	1.73 (1.47, 2.04)		<0.001	
Severe mental illness	2.16 (1.92, 2.43)	<0.001	1.31 (1.13, 1.52)		<0.001	
MI/Coronary artery disease	1.01 (0.91, 1.11)	0.91	1.19 (1.06, 1.34)		0.003	
Congestive heart failure	2.92 (2.41, 3.54)	<0.001	2.38 (1.91, 2.97)		<0.001	
Diabetes mellitus	1.27 (1.17, 1.39)	<0.001	1.17 (1.05, 1.31)		0.004	
Hypertension	0.94 (0.87, 1.02)	0.15	1.02 (0.91, 1.14)		0.71	
Peripheral vascular disease	1.28 (1.08, 1.51)	0.004	1.09 (0.90, 1.32)		0.40	
Pulmonary disorder/COPD	2.03 (1.81, 2.28)	<0.001	1.69 (1.48, 1.93)		<0.001	
Stroke/TIA	1.61 (1.35, 1.94)	<0.001	1.55 (1.26, 1.91)		<0.001	
Hyperlipidemia	0.76 (0.70, 0.82)	<0.001	0.82 (0.75, 0.91)		<0.001	
Flu	1.56 (1.44, 1.70)	<0.001	1.08 (0.98, 1.20)		0.10	
Obesity	1.25 (1.12, 1.39)	<0.001	0.97 (0.85, 1.10)		0.61	
**Primary care user level**						
High (≥3 visits/year)^c^	1.00	-	1.00		-	
Low (<3 visits/year)	0.24 (0.22, 0.26)	<0.001	0.27 (0.24, 0.29)		<0.001	
**Outside insurance**	0.62 (0.57, 0.67)	<0.001	0.87 (0.77, 0.99)		0.03	
**Service connection (%)**						
>75^c^	1.00	-	1.00		-	
51–75	0.78 (0.63, 0.96)	0.02	0.83 (0.65, 1.04)		0.10	
<50	0.48 (0.42, 0.55)	<0.001	0.68 (0.58, 0.80)		<0.001	
**Service period**						
Vietnam War^c^	1.00	-	1.00		-	
Other^d^	0.93 (0.70, 1.24)	0.62	1.14 (0.83, 1.57)		0.43	
Korean War	0.62 (0.54, 0.71)	<0.001	1.01 (0.84, 1.22)		0.89	
Persian Gulf War	1.13 (0.98, 1.31)	0.09	0.99 (0.76, 1.28)		0.92	
Post-Korean War Period	0.59 (0.50, 0.70)	<0.001	0.89 (0.73, 1.09)		0.27	
Post-Vietnam Period	1.68 (1.45, 1.94)	<0.001	1.36 (1.13, 1.64)		<0.001	
World War II	0.77 (0.68, 0.88)	<0.001	1.39 (1.11, 1.75)		0.004	
**Assigned provider(s)**						
PCP only^c^	1.00	-	1.00		-	
Trainee & PCP	1.24 (1.13, 1.37)	<0.001	0.78 (0.69, 0.89)		<0.001	
**Zip code distance from ED**						
≤2^c^	1.00	-	1.00		-	
3–5	1.10 (0.95, 1.27)	0.19	1.06 (0.90, 1.25)		0.49	
6–10	0.87 (0.62, 1.24)	0.45	1.10 (0.75, 1.61)		0.63	
≥11	0.53 (0.48, 0.59)	<0.001	0.58 (0.51, 0.65)		<0.001	

Abbreviations: ED, emergency department; PY, per-year; CI, confidence interval; IQR, interquartile range; OR, odds ratio; AOR, adjusted odds ratio; COPD, chronic obstructive pulmonary disease; TIA, transient ischemic attack; PTSD, post-traumatic stress disorder; ED, emergency department; PCP, primary care provider.

a The adjusted model excluded 1289 (9.6%) patients because they were missing one or more variables in the model.

b Continuity of care levels were excluded because of potential for colinearity with the main outcome of interest, overall continuity.

c Reference value.

d Because of the small number of veterans in these groups, they were combined: active military personnel, CAV/NPS, ChampVA spouse and children, non-Veteran humanitarian groups, merchant marines, and Tricare.

We evaluated the association of different continuity levels and ED utilization amongst individuals having at least one primary care visit with continuity – low (2%), medium (10%), and high (88%). Those with low continuity had an ED visit rate of 662/1000 PY (95% CI: 407, 1076), compared to patients with medium continuity at 585/1000 PY (95% CI: 298, 1148; p = 0.20) and high continuity at 533/1000 PY (95% CI: 276, 1031; p = 0.01) (**Table 4**). Analyzing odds ratios, individuals with medium and high levels of continuity were 31% less likely (AOR 0.69; 95% CI: 0.54, 0.92) and 41% less likely (AOR 0.59; 95% CI: 0.25, 0.76) to go to the ED compared to those with low continuity. Evaluating those individuals with low (<3 visits/year) versus high (≥3 visits/year) primary care users, those with low usage were 73% less likely (AOR 0.27; 95% CI: 0.24, 0.29) to use the ED.

**Table 4:** Association Between Levels of Continuity of Care and Emergency Department Utilization for Patients with ≥1 Continuity Visit.

Adjusted Rates of ED Utilization^a^		
Variables	Visits/1000 PY (95% CI)	p-value
**Continuity of care level**		
Low (<33%)^b^	662 (407, 1076)	-
Medium (33–50%)	585 (298, 1148)	0.20
High (>50%)	533 (276, 1031)	0.01
**Adjusted Odds of ED Utilization** a		
**Variables**	**AOR (95% CI)**	**p-value**
**Continuity of care level**		
Low (<33%)^b^	1.00	
Medium (33–50%)	0.70 (0.54, 0.92)	<0.001
High (>50%)	0.59 (0.25, 0.76)	<0.001
**Age/10**	0.91 (0.84, 0.99)	0.02
**Male**	0.96 (0.78, 1.20)	0.74
**Comorbidities**		
Smoker	1.24 (1.10, 1.39)	<0.001
Substance use disorder	1.74 (1.48, 2.04)	<0.001
Severe mental illness	1.30 (1.12, 1.51)	<0.001
MI/Coronary artery disease	1.19 (1.06, 1.34)	0.003
Congestive heart failure	2.36 (1.89, 2.95)	<0.001
Diabetes mellitus	1.18 (1.06, 1.31)	0.003
Hypertension	1.03 (0.92, 1.15)	0.63
Peripheral vascular disease	1.08 (0.89, 1.31)	0.41
Pulmonary disorder/COPD	1.68 (1.47, 1.92)	<0.001
Stroke/TIA	1.54 (1.26, 1.90)	<0.001
Hyperlipidemia	0.83 (0.75, 0.92)	<0.001
Flu	1.09 (0.99, 1.20)	0.08
Obesity	0.96 (0.84, 1.09)	0.49
**Primary care user level**		
High (≥3 visits/year)^b^	1.00	
Low (<3 visits/year)	0.28 (0.26, 0.32)	<0.001
**Outside insurance**	0.87 (0.77, 0.98)	0.02
**Service connection (%)**		
>75%^b^	1.00	
51–75%	0.82 (0.65, 1.04)	0.10
<50%	0.69 (0.59, 0.80)	<0.001
**Service Period**		
Vietnam War^b^	1.00	
Other^c^	1.14 (0.82, 1.57)	0.44
Korean War	1.00 (0.83, 1.21)	0.97
Persian Gulf War	0.97 (0.75, 1.26)	0.85
Post-Korean War Period	0.89 (0.73, 1.09)	0.25
Post-Vietnam Period	1.34 (1.12, 1.61)	0.002
World War II	1.39 (1.10, 1.74)	0.005
**Assigned provider(s)**		
PCP only^b^	1.00	
Trainee & PCP	0.82 (0.72, 0.93)	0.002
**Zip code distance from ED**		
≤2^b^	1.00	
3–5	1.06 (0.90, 1.25)	0.48
6–10	1.09 (0.74, 1.59)	0.67
11–50	0.55 (0.47, 0.64)	<0.001
>50	0.59 (0.52, 0.66)	<0.001

Abbreviations: ED, emergency department; PY, person-year; CI, confidence interval; IQR, interquartile range; OR, odds ratio; AOR, adjusted odds ratio; COPD, chronic obstructive pulmonary disease; TIA, transient ischemic attack; PTSD, post-traumatic stress disorder; ED, emergency department; PCP, primary care provider.

a The adjusted model excluded 1289 (9.6%) patients because they were missing one or more variables in the model.

b Reference value.

c Because of the small number of veterans in these groups, they were combined: active military personnel, CAV/NPS, ChampVA spouse and children, non-Veteran humanitarian groups, merchant marines, and Tricare.

### Stratified Analysis, Sensitivity Analysis, Interactions

To further evaluate the independence of the association between continuity and ED utilization, we calculated stratum-specific odds ratios for the number of primary care clinic visits during the study period instead of dichotomizing the population into “low” and “high” primary care users. These models, adjusted for the same covariates in Table 3, found a similar association between continuity and ED visits (**Table 5**) for patients who had one visit (AOR 0.41; 95% CI: 0.24, 0.68) and two visits (AOR 0.39; 95% CI: 0.25, 0.61) in the year. However, the association between continuity on ED utilization did not reach statistical significance when looking at individuals with three, four, or five or more visits in the year.

**Table 5:** Association Between Continuity and Emergency Department Utilization, Stratified by Total Number of Primary Care Visits.

No. of Primary Care Visits	AOR (95% CI)*	p-value
1	0.41 (0.24, 0.68)	<0.001
2	0.39 (0.25, 0.61)	<0.001
3	1.08 (0.52, 2.23)	0.84
4	1.54 (0.55, 4.35)	0.41
5+	1.09 (0.49, 2.40)	0.84

AOR, adjusted odds ratio; CI, confidence interval.

*The adjusted odds ratio is the association between continuity and emergency department utilization based on the same covariates used in the model as outlined in Table 3.

Because of the high proportion of patients with >50% of their visits with continuity of care, the high continuity individuals were further categorized into patients with >75% of their visits having continuity with their assigned PCP (data not shown). These patients were 51% less likely to utilize the ED compared to patients with low continuity (AOR 0.49; 95% CI: 0.38, 0.64).

## Discussion

This study found continuity of care had an independent association with lower ED utilization after controlling for age, gender, patient comorbidities, primary care user levels, having outside insurance, service connection status, military service period, distance from the VA, and having an associate provider. Having a trainee manage the outpatient care of a patient was associated with higher ED utilization in the unadjusted model (AOR 1.24; 95% CI: 1.13, 1.37), but was then associated with lower ED utilization in the adjusted model (AOR 0.78; 95% CI: 0.69–0.89), illustrating there was confounding.

To our knowledge, this is the first detailed description of PCMH continuity of care and an analysis of the association between continuity and ED utilization within this model, an important downstream outcome for PCMH care [14]. A Canadian study has evaluated the association between continuity and ED utilization within the large integrated Canadian health system, which showed that no continuity was associated with a 27 to 49% increase in ED usage [15]. These results are difficult to extrapolate, however, given the multiple differences between the VA and the Canadian health system that may confound the effects of continuity. Gill et al. measured continuity prior to the widespread uptake of PCMH models, evaluating Delaware’s Medicaid data from 1993–1994 using multivariable analyses, showing an 18% decrease in the odds of having a single ED visit and a 35% decreased odds of having multiple ED visits in a year among individuals with 3 or more primary care visits in a year [16].

To date, most reports evaluating the impact of PCMH models have focused on studying the methodology of implementing and integrating PCMH models into pre-existing medical practices [18], [28], [29]. Some studies suggest that PCMH models might improve continuity, but conclusions are based on perceptions of continuity, not continuity indices [30]–[32]. One study by Rosland et al. does report significantly improved continuity from 80% in December 2009 to 83% in June 2012 across all VA’s nationally after the implementation of PACT [12]. However, because the implementation of PACT is not expected to be complete at all VA’s until 2014, this analysis cannot fully characterize continuity across all centers and it does not analyze the association between continuity and ED use. Moreover, our methodology and dataset allows us to evaluate the impact of trainees. Large analyses have not been able to delineate continuity at the level of the trainee, while studies of trainees working within PCMH models describe qualitative experiences, not quantitative outcomes such as continuity or healthcare utilization patterns [12], [30], [31], [33]. Understanding the impact of trainees is also important because corollaries exist between residency outpatient clinics and non-training, interprofessional clinics where a physician supervises NP’s and PA’s.

Our study has limitations, notably unmeasured confounders that are inherent limitations to retrospective, observational studies. First of all, we cannot fully account for all the PACT-related components implemented in the years prior to the formalized implementation of PACT teams in 2011 at the WHVA because of the irregular nature and timing of systems-based changes required for PACT implementation. Additionally, given the VA’s work-environment culture of enhancing system performance, continuous quality improvement cycles were occurring throughout the study period affecting our ability to isolate and characterize systems changes that were fluid in nature. We can report that during the study period, major interventions for improving continuity, for example open-access scheduling or weekend clinic days, were not implemented at the WHVA. Moreover, we attempted to control for variability in PACT implementation by isolating our study to the WHVA, while an analysis of multiple facilities or large regional health systems might not be able to control for accurately. Secondly, our study design did not allow us to address the quality of a visit or account for education provided to patients on how to appropriately use the primary care clinic or the ED. Thirdly, we did not capture visits to other members of the health teams as defined in other PCMH models – pharmacists, physical therapists, social workers, mental health providers, medical or surgical specialty services, or medical assistants, who might have contributed to patient care and potentially prevented ED visits. The frequency of patient encounters with registered nurses, a key component of PACT teams, was recorded but not included in the analysis because only two percent of primary care clinic visits were exclusively with registered nurses (data not shown) and were therefore unlikely to alter our conclusions. Fourth, we cannot account for ED visits outside of the WHVA system or for patient preference to go to the WHVA ED or not. We attempted to control for factors known to influence a patient’s selection to visit one ED over another by accounting for the presence of outside insurance, the level of service connection to the VA, and the distance that a patient lived from the WHVA ED [34], [35]. Fifth, our analysis does not explore how PACT changed continuity at the WHVA and whether or not changing continuity, as PCMH aims to improve, altered ED utilization. Sixth, though we controlled for patient-level data in the adjusted model, there might be other unmeasured variables that are unavailable for a dataset such as ours. For example, individuals with less than three visits in a year, “low” primary care users, were less likely to visit the ED. Patients using the primary care clinic less might have more flexible personal schedules to see their provider because they were less sick. In contrast, those who visited the clinic three or more times in the year might have needed to be seen more urgently because of their comorbidities. Therefore, they might be inclined to see any available provider, not their assigned PCP; equivalently, they might be more likely to utilize the ED to receive care. We attempted to control for these patient-level characteristics by accounting for comorbidities, but could not account for exacerbations of their comorbid conditions prompting more frequent visits. Finally, the association between higher continuity of care levels and lower risk of ED visits cannot be used to conclude that this is a causal relationship.

In conclusion, higher continuity of care within a PCMH model was associated with decreased ED utilization. Further analyses of PCMH should evaluate the magnitude of PCMH’s impact on continuity of care, how changes in continuity might alter ED utilization, and explore other arenas of healthcare utilization, including hospitalization and readmissions. Moreover, as the volume of studies on PCMH models grows, emphasis should be placed on understanding individual components or features of PCMH, allowing clinics and integrated healthcare networks to focus on specific PCMH components shown to improve the efficient use of healthcare services by patients which may potentially save costs to the US health system.
