# Correction: Continuity in a VA Patient-Centered Medical Home Reduces Emergency Department Visits

**DOI:** 10.1371/journal.pone.0106272

**Published:** 2014-08-27

**Authors:** The PLOS ONE Staff

There are errors in Table 4. The title of the table should read “Adjusted Odds of ED utilization” and the column labelled “Visits/1000 PY” should read “AOR”. The corrected table is shown below.

**Table 4:** Association Between Levels of Continuity of Care and Emergency Department Utilization for Patients with ≥1 Continuity Visit^a^.

Adjusted Rates of ED Utilization		
**Variables**	**Adjusted Rates (95% CI)**	**p-value**
Low (<33%)^b^	662 (407, 1076)	-
Medium (33-50%)	585 (298, 1148)	0.20
High (>50%)	533 (276, 1031)	0.01

Adjusted Odds of ED Utilization		
**Variables**	**AOR (95% CI)**	**p-value**
**Continuity of care level**		
Low (<33%)^b^	1.00	
Medium (33-50%)	0.70 (0.54, 0.92)	<0.001
High (>50%)	0.59 (0.25, 0.76)	<0.001
**Age/10**	0.91 (0.84, 0.99)	0.02
**Male**	0.96 (0.78, 1.20)	0.74
**Comorbidities**		
Smoker	1.24 (1.10, 1.39)	<0.001
Substance use disorder	1.74 (1.48, 2.04)	<0.001
Severe mental illness	1.30 (1.12, 1.51)	<0.001
MI/Coronary artery disease	1.19 (1.06, 1.34)	0.003
Congestive heart failure	2.36 (1.89, 2.95)	<0.001
Diabetes mellitus	1.18 (1.06, 1.31)	0.003
Hypertension	1.03 (0.92, 1.15)	0.63
Peripheral vascular disease	1.08 (0.89, 1.31)	0.41
Pulmonary disorder/COPD	1.68 (1.47, 1.92)	<0.001
Stroke/TIA	1.54 (1.26, 1.90)	<0.001
Hyperlipidemia	0.83 (0.75, 0.92)	<0.001
Flu	1.09 (0.99, 1.20)	0.08
Obesity	0.96 (0.84, 1.09)	0.49
**Primary care user level**		
High (≥3 visits/year)^b^	1.00	
Low (<3 visits/year)	0.28 (0.26, 0.32)	<0.001
**Outside insurance**	0.87 (0.77, 0.98)	0.02
**Service connection (%)**		
>75%^b^	1.00	
51-75%	0.82 (0.65, 1.04)	0.10
<50%	0.69 (0.59, 0.80)	<0.001
**Service Period**		
Vietnam War^b^	1.00	
Other^c^	1.14 (0.82, 1.57)	0.44
Korean War	1.00 (0.83, 1.21)	0.97
Persian Gulf War	0.97 (0.75, 1.26)	0.85
Post-Korean War Period	0.89 (0.73, 1.09)	0.25
Post-Vietnam Period	1.34 (1.12, 1.61)	0.002
World War II	1.39 (1.10, 1.74)	0.005
**Assigned provider(s)**		
PCP only^b^	1.00	
Trainee & PCP	0.82 (0.72, 0.93)	0.002
**Zip code distance from ED**		
≤2^b^	1.00	
3-5	1.06 (0.90, 1.25)	0.48
6-10	1.09 (0.74, 1.59)	0.67
11-50	0.55 (0.47, 0.64)	<0.001
>50	0.59 (0.52, 0.66)	<0.001

Abbreviations: ED, emergency department; PY, person-year; CI, confidence interval; IQR, interquartile range; OR, odds ratio; AOR, adjusted odds ratio; COPD, chronic obstructive pulmonary disease; TIA, transient ischemic attack; PTSD, post-traumatic stress disorder; ED, emergency department; PCP, primary care provider.

a The adjusted model excluded 1289 (9.6%) patients because they were missing one or more variables in the model.

b Reference value.

c Because of the small number of veterans in these groups, they were combined: active military personnel, CAV/NPS, ChampVA spouse and children, non-Veteran humanitarian groups, merchant marines, and Tricare.
